# Editorial: From diet to dental health: harnessing data and digital health records

**DOI:** 10.3389/froh.2025.1773659

**Published:** 2026-01-19

**Authors:** Grace Gomez Felix Gomez, Muhammad Shahzad

**Affiliations:** 1 Department of Dental Public Health and Dental Informatics, Indiana University School of Dentistry, Indianapolis, IN, United States; 2 Regenstrief Institute Inc., Indianapolis, IN, United States; 3 Faculty of Dentistry, Zarqa University, Zarqa, Jordan; 4 Institute of Basic Medical Sciences, Khyber Medical University, Peshawar, Pakistan

**Keywords:** electronic dental records, electronic health records, nutritional recommendations, oral health, precision nutrition

## Introduction

### “Miles to go” for good oral health through precision nutrition

We are not there yet, but the journey of a thousand miles has begun with the vision of achieving personalized nutrition recommendations to improve oral and general health. In this digital era, the rapid transition of paper-based health records to electronic dental (EDR) and health (EHR) records, coupled with artificial intelligence (AI) provides unprecedented opportunities for managing complex health data. Leveraging big data through machine learning (ML) approaches will enable the implementation of precision nutrition by accounting not only for dietary patterns but also for other crucial modifiable factors such as physical activity and adiposity, which have an established role in oral and systemic health (1, 2). According to the 2022 global oral health status report, an estimated 3.5 billion people are affected by non-communicable oral diseases, conditions that can adversely affects dietary intake and thereby nutritional status of individuals (3). This special Research Topic of research topics, “*From diet to dental health: harnessing data and digital health records*,” through Frontiers in Oral Health, brings together five diverse yet crucial research evidence leveraging big data to integrate nutrition and dental records for better oral and systemic health outcomes (4).

Together, EDR and EHR provide extensive information on the individual's health and dental conditions, along with their demographic, behavioral, social, lifestyle, psychological, and dietary factors. This information, through artificial intelligence-based prediction models, could be translated into evidence-based nutritional recommendations for improving oral and systemic health in a dental setting. However, extracting meaningful information from a large EHR-based dental data set not only requires careful evaluation of data quality such as checking for completeness and accuracy but also recognition of inherent limitations such as self-selection bias and potential inaccuracies in routinely collected clinical data. The BigMouth Dental Data Repository study by Tiwari et al., highlights the importance of this by evaluating half a million patient visits' EHR data for completeness. Despite the core demographic data being complete, they reported notable differences in completeness for dental and behavioral data, as well as based on provider types (students, faculty, residents) from eleven dental schools. The findings emphasize not only the need for a dental data repository itself, but also standardized data entry practices, education and training on complete and thorough data collection, and interpretation of EHR-based variables for instituting precision-based care.

Drinking water fluoridation is an important milestone in public health to prevent dental caries at the population level primarily through its topical impact on enamel by inhibiting demineralization and enhancing remineralization (5). Systemic fluorides intake especially during tooth development also enhance lifelong resistance to dental caries. In societies where water fluoridation is not implemented or inaccessible, the fluoride contents in food can provide protection against dental caries. However, aside from a few fluoride ingestion studies (6, 7) comprehensive data on the bioavailability/accessibility of fluoride-containing foods are still lacking. To address this, Kronic et al., quantified dietary fluoride of 103 commonly consumed foods by children. The mean bio accessibility of food fluoride was approximately 44% underscoring significant differences in bioavailability. Fluoride utilization through food consumption is low but increases when combined with tap water, milk, and drinks underscoring the importance of monitoring food and revisiting dietary fluoride guidelines.

Additionally, dietary intake and feeding practices also play a crucial role in the formation of dental caries among young children. In this context, an important study published in this collection by Hu et al., provides valuable insights into feeding practices, especially bedtime feeding habits and dental caries prevalence in 5–7 year-old children. These two studies further reinforce the importance of integrating dietary components into patients' dental records. These recommendations are important to establish seamless interoperability between medical and dental domains and promote a cross-disciplinary approach.

Dietary patterns also influence the connection between systemic health and oral conditions especially periodontal diseases. For examples, diets rich in carbohydrates, saturated fats and processed foods are frequently associated with a plethora of systemic conditions including diabetes, dyslipidemia and hypertension, which in turn exacerbate periodontal diseases. In contrast, a healthier diet encompassing fiber, fruits, vegetables, legumes, lean protein sources, and unsaturated fats support metabolic health and decrease periodontal disease risk (8, 9). Although, electronic dental records rarely capture dietary information, incorporating structured dietary data would help identifying at risk individuals and promote precision-based nutrition practice within a dental clinical setting. In this collection, Zhang et al., have reported how different dietary indices are associated with periodontal disease risk. Using NHANES data, they have shown that poor adherence to a particular dietary plan, e.g., DASH (Dietary Approaches to Stop Hypertension) diet, is associated with a high risk of periodontitis at the population level. Therefore, an automated system for calculating dietary indices incorporated into the EDR system will help in assessing their risk of oral diseases.

Older adults are more susceptible to oral health problems partly due to changes in taste perception associated with advancing age. In this collection, Alves et al., conducted a systematic review and meta-analysis of 18 observational studies demonstrating altered taste sensation in older adults is associated with higher consumption of sugary foods and increase prevalence of oral health problems. Moreover, tooth loss and impaired masticatory functions with advancing age forces elderly individuals to preferentially consume more softer and processed foods that leads to chronic disease conditions in older populations. Collectively, these studies highlight the lack of data elements for dietary and nutritional information within EDR, thus limiting the ability of the ML to predict oral disease outcomes. For precision nutrition, it is essential that these data elements be available in large datasets to identify patterns and relationships between diet, oral health and systemic conditions.

In summary, since precision nutrition relies on accurately collected data, appropriate education and protocols must be in place to enforce this requirement. However, it is not impossible, as machine learning and artificial intelligence could help in reverse-engineering the processing of clinical notes to curate information that could be cross-validated with the patient's health records to provide comprehensive and personalized nutritional guidance in a dental setting. The voluminous amount of data that is already collected can be evaluated for its data quality and accuracy to make informed decisions. We learn from existing data, and since not all dietary data are currently captured in health and dental records, we are on a well-learned road to achieve the goal in every step of understanding information from big data. Incorporation of dietary data variables, gathering accurate and complete data, will contribute towards predictive modeling on understanding the association of oral health diseases and its influence on overall health outcomes as it will help with integrating clinical decision support system for promoting an interdisciplinary care.
